# The Effect of Preoperative Education on Psychological, Clinical and Economic Outcomes in Elective Spinal Surgery: A Systematic Review

**DOI:** 10.3390/healthcare7010048

**Published:** 2019-03-21

**Authors:** Louise C. Burgess, Joe Arundel, Thomas W. Wainwright

**Affiliations:** Orthopaedic Research Institute, Bournemouth University, Bournemouth, Dorset BH8 8EB, UK; lburgess@bournemouth.ac.uk (L.C.B.); jarundel@bournemouth.ac.uk (J.A.)

**Keywords:** preoperative education, patient education, spine surgery, enhanced recovery after surgery (ERAS), fast-track

## Abstract

Psychosocial factors related to different degrees of clinical impairment and quality of life in the preoperative period may influence outcomes from elective spine surgery. Patients have expressed a need for individualized information given in sufficient quantities and at the appropriate time. Therefore, this review article aims to determine whether a preoperative education session improves clinical, psychological and economic outcomes in elective spinal surgery. PubMed, Cochrane Library, CINAHL Complete, Medline Complete and PsychINFO were searched in July 2018 for randomized clinical trials to evaluate the effects of a preoperative education intervention on psychological, clinical and economic outcomes in spinal surgery. The search yielded 78 results, of which eleven papers (seven studies) were relevant for inclusion. From these results, there is limited, fair-quality evidence that supports the inclusion of a preoperative education session for improving clinical (pain, function and disability), economic (quality-adjusted life years, healthcare expenditure, direct and indirect costs) and psychological outcomes (anxiety, depression and fear-avoidance beliefs) from spinal surgery. Other benefits are reported to be improved patient knowledge, feelings of better preparation, reduced negative thinking and increased levels of physical activity after the intervention. No differences in quality of life, return to work, physical indicators or postoperative complications were reported. From the limited evidence, it is not possible to conclusively recommend that preoperative education should be delivered as a standalone intervention before elective spine surgery; however, given the low risk profile and promising benefits, future research in this area is warranted.

## 1. Introduction

Spinal surgery for the treatment of back or neck pain may be considered if conservative strategies are unsuccessful, pain becomes disabling or in cases of progressive neurological deficit and deformity [1]. A recent development in spinal surgery has been the systematic application of an evidence-based perioperative care protocol (named “fast-track” or “enhanced recovery after surgery” (ERAS)) [2] which aims to attenuate the surgical stress response and reduce convalescence [3]. Although considered a very successful tool to achieve high quality standards of treatment paired with a reduction in hospital stay [4], enhanced recovery pathways have not been widely implemented within spinal procedures [3]. The perception of pain in relation to spinal surgery can be altered by external factors, and psychosocial characteristics related to different degrees of clinical impairment and quality of life in the preoperative period may influence surgical recovery [5]. For example, patients with chronic and disabling symptoms may experience symptoms of anxiety and depression, which are important outcome predictors of physical impairment, pain and lower health-related quality of life [6,7,8]. Factors associated with anxiety and depression both before and after spine surgery are reported to be a greater experience of pain, the need for information, the degree of self-reported disability, employment status (feelings of not being able to contribute to society) and mental health status [8]. The need for information is reported to be a significant theme amongst qualitative studies from spinal surgery, with patients expressing a need for individualized information given in sufficient quantities and at the appropriate time [8].

Preoperative education is a core component of ERAS pathways [2] that aims to empower patients and their families to undertake positive health actions and support autonomous decision making [9]. It has been described as providing patients with health-related information, teaching them skills aimed at reducing discomfort and complications and offering psychological support [10]. As perception can be altered by external factors [5], providing patients with pertinent information may help to attenuate preoperative anxiety by inducing feelings of control [8]. There is evidence to suggest that those who gain sufficient knowledge can improve their coping ability and subsequently engage in appropriate attitudes and behaviors [11]. In addition, patient activation, defined as one’s propensity to engage in efficacious health behaviors, has been identified as an important modifier of the recovery process, leading to better outcomes and increased compliance with physical therapy after spine surgery [12]. Given that behaviors such as catastrophizing and kinesiophobia may coexist and become important determinants of disability in this patient group [11], implementing a preoperative education intervention may encourage adaptive coping strategies ahead of spine surgery. The effect of prehabilitation (enhancing functional capacity) on functional and economic outcomes from spinal surgery has recently been investigated [1]; however, to date, no review has sought to summarize the specific effect of an education intervention. Therefore, the purpose of this review is to determine whether a preoperative education intervention improves psychological, clinical or economic outcomes from elective spinal surgery. The research question has been described using the PICOS (population, intervention, comparison, outcome, study) criteria in Table 1.

## 2. Materials and Methods

This study was developed according to the Preferred Reporting Items for Systematic Reviews and Meta-Analyses (PRISMA) statement [13]. A literature search was completed in July 2018, and the electronic databases sourced included PubMed, Cochrane Library, CINAHL Complete, Medline Complete and PsychINFO, accessed through Bournemouth University’s online library.

A search strategy was developed to capture articles that had compared a preoperative education intervention to standard preoperative care ahead of elective spinal surgery (Table 2). Studies were included if they were conducted in cohorts of adults (aged 18 years or over) receiving surgery, or revision surgery, on their lumbar, cervical, thoracic or sacrum region of their spine and compared a preoperative education or counselling intervention to standard pre-surgery care. Studies that included spinal surgery for the treatment of scoliosis, kyphosis or lordosis were also included. Studies that included spinal surgery for children (aged less than 18 years) and on the coccyx region of the spine were excluded from the search.

The search reviewed all fields of the available peer-reviewed literature published in the English language (or where a translation was available) from the earliest record on file until July 2018. Studies were only included if they were randomized clinical trials, in order to produce conclusions untainted by bias. The predetermined eligibility criteria are listed in Table 1. Multimodal prehabilitation programmes that include physical, nutritional and psychological components are reported to be the most effective [14], and thus preoperative interventions with a number of ingredients were included in our results, although it was acknowledged that the results of these studies could not be solely attributed to the educational component. Studies were included if their outcome measures were clinical (for example, pain, disability, length of stay), psychological (for example, anxiety, depression or fear avoidance beliefs) or cost-related (economic evaluations), recorded either after intervention (pre-surgery) or at any time point post-surgery. Where available, differences in outcome measure means were the primary summary measure.

Once records were identified through database searching, duplicates were removed, and then the titles and abstracts of the remaining articles were screened for eligibility by two reviewers (L.C.B. and J.A.). Following the removal of irrelevant articles, full-text screening was conducted by two reviewers (L.C.B. and J.A.). Any discrepancies between reviewers were resolved through discussion.

### Quality Assessment

The Cochrane Collaboration’s tool for assessing the risk of bias in randomized trials was used to evaluate the methodological quality of the articles included within our review. The tool includes six variations of bias: selection bias, performance bias, detection bias, reporting bias and other bias [15]. Two authors (L.C.B. and J.A.) completed the risk-of-bias assessment and resolved any discrepancies through discussion. The assessment of the risk of bias was made for the study as a whole (study-level), rather than for each outcome measure, as recommended by the Cochrane Handbook [16].

## 3. Results

**Figure 1:**
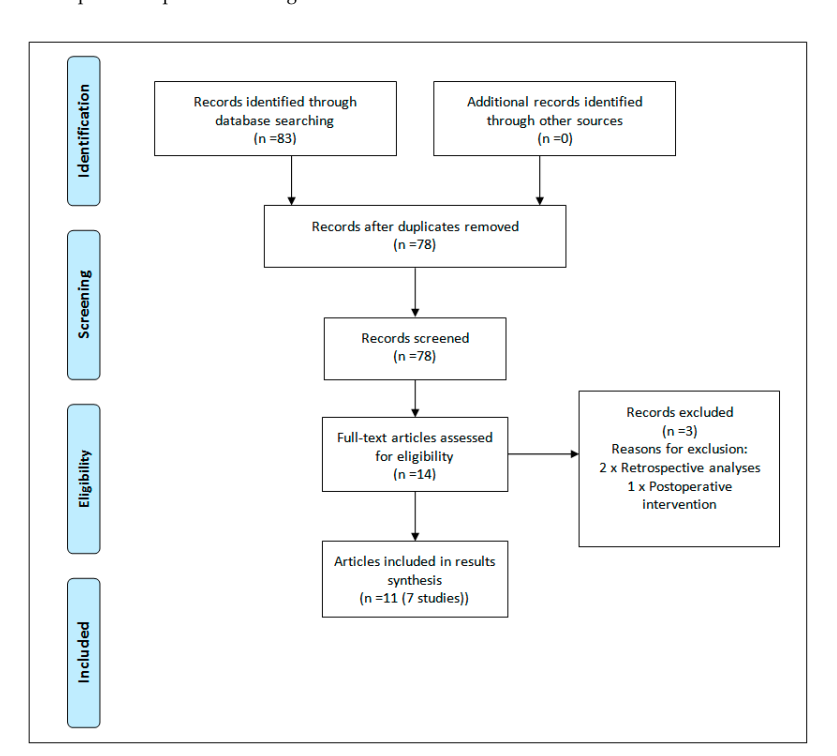
Preferred Reporting Items for Systematic Reviews and Meta-Analyses (PRISMA) flowchart [13].

The search generated 83 results, which was reduced to 78 once duplicates were removed. Titles and abstracts of the remaining studies were screened for eligibility, with 64 irrelevant texts removed. Fourteen papers underwent full-text screening, with a further three articles removed. Two papers were removed as they were retrospective analyses [17,18] and one screened patients preoperatively but did not deliver the educational intervention until six weeks after surgery [19]. Secondary searching was undertaken, whereby reference lists of the selected articles were reviewed for additional studies not identified in the primary search, but did not uncover any additional results. The flowchart of the study selection process is presented in Figure 1.

The search yielded seven studies (11 papers) (664 participants) that investigated the effect of preoperative education interventions on psychological, clinical and economic outcomes following elective spine surgery (Table 3) [20,21,22,23,24,25,26,27,28,29,30]. One study included patients scheduled for decompressive lumbar surgery [23], one for lumbar spinal fusion [20], one for anterior cervical discectomy and fusion [30] and two for a combination of lumbar decompression and fusion surgeries [25,29]. Two studies did not specify a procedure but included patients undergoing surgery for the treatment of spinal stenosis [27] or for a degenerative lumbar spine disorder, the presence of low back or leg pain because of disc herniation, spinal stenosis, spondylolisthesis (grades 1–2) or degenerative disc herniation [26]. Two interventions [25,26] were multimodal in nature, with one study combining preoperative physiotherapy, education, a supplement drink and early rehabilitation [25] and the other including physiotherapy, an individualized exercise programme and a behavioral approach to fear avoidance and physical activity [26]. Therefore, it should be acknowledged that the conclusions from these studies cannot solely be attributed to the educational component of the intervention.

### 3.1. Methodological Quality

Methodological quality scores for each study as a whole are provided in Table 4. One study was scored as “high quality” [28] and the remaining as “fair quality” [20,23,25,26,29,30]. Consistently low scoring items included the blinding of participants and personnel, blinding of outcome assessment and other bias. However, due to the nature of the intervention delivered, it was not always possible to blind the patient and clinician, and therefore no study was considered “poor quality”, which would suggest an important methodological limitation that could invalidate results [15].

### 3.2. Clinical Outcomes

Pain was an outcome measure in seven of the 11 articles included in this review [20,21,23,25,26,28,29]. Louw et al. [23] found no difference between a preoperative neuroscience education programme and usual preoperative care for mean low back or leg pain scores at pre-surgery, one month, three months, six months and 12 months post-surgery as measured by the numeric pain rating scale (NPRS). Similarly, Rolving et al. [21] measured average daily pain from postoperative day one to seven and found no difference in median back pain severity scores between those receiving preoperative cognitive-based therapy (NPRS 5.6, range 1.7–10.0) to those on a standard care pathway (NPRS 5.3, range 1.1–7.7) (*p* = 0.73). At the 12-month follow up, Rolving et al. [20] found no significant between-group differences in the change from baseline to one year post-surgery for leg pain (intervention group (IG): −2.8 (range −4.7 to −1.3), control group (CG): −1.3 (range −6.0 to −0.3) (*p* = 0.73)) or back pain (IG: −2.5, range −4.3 to −1.0, CG: −2.7, range −5.0 to −0.3) (*p* = 0.23)) as measured by the low back pain rating scale. Likewise, in the study by Kesanen et al. [28] although visual analogue pain scores (VAS) for back and leg pain decreased during follow up for patients from baseline to follow up, there were no differences between those with receiving a preoperative feedback session and those receiving standard care.

In contrast, the benefits of preoperative education on pain scores are reported in three studies [25,26,29]. Patients who received a preoperative educational intervention for anxiety and pain the day before surgery reported lower mean pain scores using VAS both 30 min before surgery (IG VAS (mean ± standard deviation (SD)): 7.14 ± 1.00, CG VAS: 7.60 ± 0.78 (*p* = 0.024)) and on the day after surgery (IG VAS: 5.36 ± 0.82, CG VAS: 6.28 ± 0.91) (*p* < 0.001)) [29]. Similarly, Nielsen et al. [25] report significantly less pain (as measured as area under curve (*p* = 0.03) and less low back pain intensity (area under curve (*p* = 0.02)) for patients receiving prehabilitation and early rehabilitation compared to standard care, although there were no differences in the average or worst low back pain or all kinds of radiating pain between groups at the six-month follow up. Lindback et al. [26] report a reduction in VAS back pain at pre-surgery for patients receiving preoperative physiotherapy compared to standard care (mean between group change −6.0, range −11.8 to −0.3 (*p* < 0.040)), but no differences were reported for leg pain pre-surgery or leg and back pain post-surgery.

Function and/or disability was assessed using the Roland Morris questionnaire [25], the Oswestry disability index (ODI) [20,23,26,28], the sit-to-stand test [25], the timed up and go test [25] and the cumulated ambulation Score [21]. Lindbäck et al. [26] report an improved function from baseline to presurgery for patients receiving presurgery physiotherapy (IG: ODI mean change (standard error (SE)): −3.2 (1.1), CG: −0.6 (1.1) (*p* = 0.27) but not at three or 12 months post-surgery. Median self-reported function was reported to improve at hospital admission for patients who received a prehabilitation intervention compared to standard care (Roland Morris questionnaire score of 14 (range 1–21) versus 17 (range 7–23) respectively (*p* = 0.001)) [25]; however, no between-group differences were recorded for timed up and go or sit to stand scores [25]. Despite this, Nielsen et al. [25] report a reduced length of hospital stay for the intervention group (median (range): 5 (3–9) days) compared to the control group (7 (5–15) days (*p* = 0.007)).

Rolving et al. [21] report superior postoperative mobility as assessed by the cumulated ambulation Score for patients who received preoperative cognitive-behavioral therapy compared to a control group, and thus independent walking ability was achieved by more patients on day two (39% versus 16% respectively (*p* < 0.0026)) and on day three (73% versus 48% (*p* < 0.02)). The authors also report a better performance (median score) for getting in and out of bed (IG: 58% versus CG 26% (*p* = 0.017)) and for getting up from a chair (58% versus 26% (*p* = 0.017)) on the third day following surgery [21]. At the one year follow up, there were no differences between groups’ improvement in ODI; however, the authors report a reduction in disability at three months (−15 points, range −26 to −4 (*p* = 0.003)) for the intervention group, but no significant reduction for the control group at any of the follow ups [20]. Likewise, no differences were found between intervention and control groups for self-reported function following a preoperative neuroscience education programme [23] or a feedback session based on a knowledge test [28].

### 3.3. Psychological Outcomes

Anxiety was measured in four studies by using the hospital anxiety and depression scale (HADS) [26] and Spielbergér’s state trait anxiety inventory (STAI) [28,29,30]. Anxiety was reported to be lower 30 min before surgery (IG STAI (mean ± SD): 48.52 ± 6.24, CG: 53.75 ± 8.45 (*p* = 0.001)) and on the day after surgery (IG STAI: 45.19 ± 6.66, CG: 49.18 ± 6.81 (*p* = 0.009)) following educational intervention [29]. Similarly, a decrease in anxiety scores (5.1 points on STAI (95% Confidence Interval (CI) 0.7–9.5, (*p* = 0.011)) was reported from baseline to hospital admission following intervention, with further decreases noted after surgery, whereas in the control group, anxiety reduced only after surgery (4.8 points on STAI (95% CI 0.1–9.7, (*p* = 0.044)), although the differences between groups were not significant [28]. Chuang et al. [30] report greater decreases in anxiety and uncertainty (as measured by the Mishel uncertainty in illness scale (MUIS)) both one day before surgery (IG STAI (mean change ± SD): 12.28 ± 7.16, CG: 4.47 ± 4.52 (*p* < 0.001), IG MUIS: 19.56 ± 12.89, CG: 11.00 ± 7.42 (*p* < 0.001)) and two days after surgery (IG STAI: 19.19 ± 9.49, CG: 8.53 ± 6.34 (*p* < 0.001), IG MUIS: 22.31 ± 13.17, CG: 15.53 ± 9.19 (*p* < 0.001)). In contrast, no changes in mean anxiety (as measured by HADS) were reported between intervention and control groups following a presurgery physiotherapy and education intervention [26].

### 3.4. Fear-Avoidance Beliefs

The fear avoidance beliefs questionnaire (FABQ-PA) was used to quantify fear avoidance beliefs about physical activity in two studies [20,26]. Between-group differences were reported in favor of the education group after the intervention (IG FABQ-PA (mean change from baseline (SE): −2.5 (0.5), CG: −0.8 (0.6) (*p* = 0.14) [26], at six months following surgery (IG FABQ-PA (median change from baseline (interquartile range)): −3.0 (−5.0 to 1.0), CG: 0.0 (−2.0 to 3.0) (*p* = 0.01)) [20], but not at three months post-surgery [20] or at the one year follow up [20,26].

Other benefits of a preoperative education intervention ahead of spine surgery were reported to be improved patient knowledge at hospital admission, on the day before discharge and at three and six months after surgery [27], feelings of better preparation and surgery meeting expectations [23], increased satisfaction with care [30], less analgesic consumption (only significant on postoperative day two) [21], less health-care utilization post-surgery [23], reduced negative thinking in relation to pain at six months (but not at three months or one year post-surgery) [20], improved self-efficacy and depression [26], and higher physical activity levels after intervention [26]. No differences in returning to work [20], postoperative complications or adverse events [25] or physical indicators (blood pressure, heart rate, respiration rate and cortisol level) 30 min before surgery or on the day after surgery [29] were reported. Quality of life was reported to improve following spine surgery [24,25,28]; however, no between-group differences were observed.

### 3.5. Economic Outcomes

Economic evaluations conducted alongside these studies found favorable results for patients receiving a preoperative education intervention. The cost-effectiveness of preoperative cognitive behavioral therapy [22] was measured primarily by quality-adjusted life years (QALY) based on the EuroQol five dimensions questionnaire (EQ-5D) scores, and at one-year follow up was significantly better for the intervention group (IG QALY (mean (95% CI)): 0.710 (0.670, 0.749), CG: 0.636 (0.573, 0.687 (*p* = 0.045)); however, the length of stay in hospital was not shorter for the intervention group [21].

One year after the implementation of a neuroscience education programme ahead of lumbar radiculopathy [23], the mean healthcare expenditure for the intervention group was 45% less than the control group (IG mean expenditure ± SD: $2678.57 ± $3135.30, CG: $4833.48 ± $3256.00 (*p* = 0.007)), with patients receiving standard care using more radiographs (47 vs. 17, *p* = 0.015) and physiotherapy sessions (394 vs. 113, *p* < 0.001) than the neuroscience education group.

Nielsen et al. [24] collected data on the cost of a prehabilitation and early rehabilitation intervention preoperatively, during hospitalization and postoperatively by estimating the cost from a multiplication of the resource consumption and price per unit. The intervention group was estimated to have a €1625 lower cost (€494 direct and €1131 indirect) than the standard care programme largely due to fewer days lost, decreased hospital stay and no secondary surgery. Higher preoperative costs were recorded in the intervention group due to physical therapy evaluation and treatment, smoking intervention and pain treatment; however, this evaluation does not describe the costs of a preoperative education intervention alone. In addition, it is not possible to determine if the secondary surgery complication in the control group was due to the smoker that did not take part in the study’s smoking cessation programme or to an unrelated cause.

## 4. Discussion

Preoperative education pertains to a number of interventions that occur ahead of surgery to prepare patients for increased physical and psychological demands [17] and has previously been reported as unsatisfactory [31]. Currently, the evidence base for educational interventions ahead of spine surgery is limited by the small number of studies, their relatively small sample sizes and the diversity in surgeries before which they are delivered. The heterogeneity of the included educational interventions also prevents recommendations for future preoperative care being drawn with confidence. The interventions included within this review were cognitive-behavioral therapy [20,21], an empowering telephone call [27,28], a patient education booklet [29], an iPad and information booklet [30], a one-to-one neuroscience education programme delivered verbally with the use of pictures, examples, metaphors and drawings, and followed by an education booklet [23], and two multimodal interventions, with one intervention combining preoperative physiotherapy, education, a supplement drink and early rehabilitation [24,25] and the other including physiotherapy, an individualized exercise programme and a behavioral approach to fear avoidance and physical activity [26].

Contrasting results are presented for the effects of a preoperative education programme on both self-reported and performance-based clinical outcomes, including pain, disability and function, and the majority of the evidence base does not report a sustained benefit. Economic evaluations are lacking; however, it has been suggested that a preoperative education intervention may offer cost benefits to future healthcare expenditure [23,24] and to QALY following spine surgery [22], although a number of factors could influence the total cost of future healthcare expenditure. The results are more favorable within psychological outcome measures, particularly at pre-surgery and in the early acute recovery phase, where levels of anxiety [28,29,30], depression and self-efficacy [26] are reported to improve when compared to standard preoperative care. In addition, patients have reported feeling better-prepared for surgery [23,30] and having their expectations met [23]. These results are supported by analyses of retrospective data that report benefits of preoperative education to patient satisfaction [17,18], especially in terms of pain management [17], and postoperative back pain and number of visits to the emergency room in the 12 weeks following spine surgery [18]. These results are also similar to findings from hip and knee replacement surgeries (in which an enhanced recovery or fast-track framework is better established [32,33,34,35,36]) that support preoperative education for improving anxiety [37,38,39]. However, the interventions included within our review were not implemented as part of an ERAS or “fast-track” protocol, and we do not know of the differences in perioperative care that may have also influenced surgical outcomes. Although total hip and knee replacement have been widely accepted as cost and clinically effective procedures, spine surgery for the treatment of degenerative conditions does not always share the same perception [40,41]. Therefore, a preoperative education intervention, delivered as part of an ERAS or “fast-track” protocol, may have a greater effect on outcomes from elective spine surgery than the interventions included within this review.

Spine surgery may be perceived to have potentially uncertain outcomes, with negative side effects and a considerable recovery period [42]. Psychological factors are a commonly investigated predictor of recovery from surgery, and although their overall importance still remains equivocal [6], it is largely agreed that procedure-related uncertainty and unrealistic expectations of outcome can contribute to preoperative anxiety, which can negatively affect postoperative recovery [43]. Given that the quantity and timing of information mediates the association between risk factors and anxiety and depression [8], our results support the implementation of a preoperative education session ahead of spinal surgery. Anxiety is adaptive in motivating behavior that helps patients cope with threatening situations [44], such as surgery, and as feelings of control encourage anxiety to become facilitative [45], it appears important that patients receive sufficient preoperative information in order to improve their coping ability. However, the amount of information required to be facilitative is dependent upon the patient’s coping style [46]. For example, patients who exhibit the most denial and highest anxiety may benefit from education interventions, whereas patients directly expressing desire for information may only exacerbate their anxiety upon receiving it [38].

In such case, a preoperative identification process followed by an appropriate intervention may enhance the effects of preoperative education on postoperative recovery [47]. As there are many conceptual models of psychological preparation, the preoperative identification of risk factors is important to establish what educational intervention is required. Empowering patients through educational interventions has previously been divided into the following areas: biophysiological, function, cognitive, social, experiential, ethical and financial [48]. However, instead of addressing each of these areas, it may be more beneficial to attend to a patient’s individual psychological state and level of preparation before surgery [38] and subsequently prescribe tailored therapy. Patient-centred care is fundamental to the mission of healthcare and enhanced recovery pathways, yet traditionally patients have not been involved as partners in shaping their health services [49]. Effective communication between clinician and patient is thought to enhance compliance with treatment recommendations [50], satisfaction with care [51], overall treatment outcomes [52,53] and psychological well-being [51]. In addition, encouraging shared decisions may empower patients to become active participants in their own post-operative recovery and help to manage expectations of postoperative pain [54].

Preoperative screening may also assist in identifying highly anxious patients who catastrophize, suffer from kinesiophobia or engage in negative self-talk using validated tools such as the fear avoidance belief questionnaire, the hospital anxiety and depression scale or Spielberger’s state trait anxiety inventory, as cited within our review. For these patients, a psychologist delivering a preoperative intervention could help to reshape conceptions about the surgical and recovery process [46]. Often, it is not a situation that causes a specific emotional response, but instead an individual’s thoughts or cognitive processes regarding the task [55]. These thoughts can influence the behaviors a patient engages in, but are fortunately susceptible to change [55]. Once negative automatic thoughts have been identified, cognitive preparation for surgery can help to challenge and reframe them. Cognitive-behavioral approaches used in preparation for spine surgery generally include cognitive restructuring and deep relaxation training [46], as encouraging negative cognitions to become more positive or coping orientated can improve a patient’s ability to cope with surgery, and thus enhance outcomes. In our review, we found that Rolving et al. [20] covered topics on the interaction of cognition and pain perception, coping strategies, pacing principles, ergonomic directions, returning to work and details about the surgical procedure during their cognitive-behavioral intervention. Similarly, Lindback et al. [26] combined a behavioral approach (goal setting, strategies to minimize barriers to goal attainment and self-mediated home exercise and physical activity) with physiotherapy and a tailor-made general supervised exercise programme in an attempt to better prepare patients for surgery.

## 5. Limitations

Our review is limited to studies published in the English language and therefore may have omitted relevant articles published in a different language. The variation in education intervention delivered and the inclusion of two multimodal interventions [25,26] prevents assurances that one intervention is better than another. In addition, as previously highlighted, the results from the multimodal interventions cannot be solely attributed to the educational component. Due to the limited studies yielded, and the range of spinal procedures included within our results, we cannot offer procedure-specific results. Study-specific limitations also occur whereby possible contamination bias [27,28], between-group differences at baseline [26], a lack of control over rehabilitation [23] and poor compliance with interventions [20,21] are reported. It should also be highlighted that we do not know of the differences in perioperative pathways or other social determinants that may also affect outcomes; for example, pain levels may be affected by analgesics or anaesthesia, function by different physiotherapy routines and anxiety by different home or hospital settings. Finally, patient-reported outcomes do not always correlate with objective outcome measures [56], and therefore it is difficult to measure the accuracy of the outcome tools used to assess both clinical and psychological variables within our review.

## 6. Conclusions

This review is the first to compare the effects of a preoperative education intervention on psychological, clinical and economic outcomes from spinal surgery. The majority of results on clinical outcomes do not report a sustained benefit, and economic evaluations are lacking. From the limited evidence, it is not possible to conclusively recommend that preoperative education should be delivered as a standalone intervention; however, the importance of psychological optimization before spine surgery remains unequivocal. Given the low risk profile and the promising benefits for psychological outcome measures including anxiety, depression, fear-avoidance beliefs and self-efficacy, particularly at pre-surgery and in the acute phase of recovery, future research in this area is warranted.

## Figures and Tables

**Table 1:** PICOS criteria.

PICOS Item	Inclusion Criteria	Exclusion Criteria
Population	Adults (18 or over) receiving spine surgery. Revision spine surgery. Surgery for scoliosis, kyphosis or lordosis. Lumbar, cervical, thoracic or sacrum surgery.	Spine surgery on children (under 18). Coccyx surgery.
Intervention	Preoperative education/counselling programmes. Multimodal preoperative interventions with an educational component.	Preoperative physiotherapy alone.
Comparison	Standard preoperative care.	
Outcome Measures	Clinical (self-reported and performance based). Psychological. Economic evaluations.	
Study Design	Randomized clinical trials.	Review articles. Case studies. Historical studies. Non-randomized clinical trials.
Publication	Published in English. Access to full text.	Unpublished studies. Study protocols.

**Table 2:** Search strategy.

Population	Intervention	Timing	Publication Type
“Spine surgery” OR “back surgery” OR “spine fusion” OR “spine stenosis” OR Spondylodesis OR “spine disease” OR “disc surgery” OR “lumbar surgery” OR “thoracic surgery” OR “cervical surgery” OR “kyphosis” OR “lordosis” OR “thoracolumbar surgery” OR “degenerative scoliosis”	(“Education”) OR (“Counseling”) OR Counselling OR Education OR “Cognitive based therapy” OR CBT OR “Psychological support” OR “Neuroscience education” OR “Prehabilitation” OR “Goal setting” OR “Goal achievement”	“Preoperative education” OR “Preoperative conditioning” OR “Preoperative interventions” OR Preoperative OR Perioperative OR “Preoperative” OR “Peri operative” OR “Pre-operative” OR Before OR Prior to OR (MM “Preoperative Period”)	“Randomized controlled trial” OR “Randomised controlled trial” OR “randomized clinical trial” OR “randomised clinical trial” OR “Controlled clinical trial” OR “clinical study”

**Table 3:** Summary of preoperative education interventions for spine surgery.

Study	Population	Intervention	Control Group	Outcomes	Main Findings
Louw et al., 2014 [23] (n = 67)	Decompressive lumbar surgery. IG: n = 32 CG: n = 35	Preoperative pain-specific neuroscience education programme in addition to usual care one week prior to lumbar surgery. Topics included the following: (1) The decision to have lumbar surgery; (2) The nervous system’s physiology and pathways; (3) Peripheral nerve sensitization; (4) Surgical experiences and environmental issues effects on nerve sensitivity; (5) Calming the nervous system; (6) Recovery after lumbar surgery; (7) Scientific evidence for the neuroscience education booklet content; (8) The opportunity to reflect and write questions to ask the surgeon prior to surgery.	Usual care regarding preoperative education.	Pain (NPRS), function (ODI), postoperative thoughts/beliefs and health care utilization post-lumbar surgery.	At 1-year follow up, there were no statistical differences between groups with regard to primary outcome measure of low back pain (*p* = 0.183), leg pain (*p* = 0.075), and function (*p* = 0.365). The IG group scored significantly better in the following categories: better prepared for lumbar surgery (*p* = 0.001); preoperative session preparing them for lumbar surgery (*p* < 0.001) and lumbar surgery meeting their expectations (*p* = 0.021). Healthcare utilization post-lumbar surgery also favored the IG (*p* = 0.007).
Rolving et al., 2015, 2016a,b [20,21,22] (n = 90)	Lumbar spinal fusion. At baseline: IG: n = 59 CG: n = 31	Six 3-h cognitive-behavioral therapy group sessions (4 sessions preoperatively and 2 sessions postoperatively). Topics included the following: (1) Interaction of cognition and pain perception; (2) Coping strategies; (3) Pacing principles; (4) Ergonomic directions; (5) Return to work; (6) Details about the surgical procedure.	Usual care regarding preoperative education.	(1) Disability (ODI), psychological variables, return to work and pain (low back pain rating scale); (2) Back pain (NPRS), mobility on day-3, analgesic use, hospitalization; (3) QALY, disability (ODI).	At 1-year follow up, there was no statistically significant difference between the IG and the CG in ODI score (*p* = 0.082). However, the IG had achieved a significant reduction of −15 points (−26, −4) already at 3 months (between group difference (*p* = 0.003), and this reduction was maintained throughout the year. There was no difference between the groups’ self-reported back pain (*p* = 0.76). Independent mobility was reached by a significantly larger number of patients in the IG than the CG during the first three postoperative days. Analgesic consumption tended to be lower in the IG, whereas length of hospitalization was unaffected by the CBT intervention. One year after the surgery, the estimated QALY was significantly better for the IG. There was no difference in the overall costs of the two groups.
Nielsen et al., 2008, 2010 [24,25] (n = 60)	Spinal surgery for degenerative disease with low back pain and radiating pain (decompression and fusion). Received intervention: IG: n = 28 CG: n = 32	Prehabilitation and early rehabilitation, for 30 min daily for 6 to 8 weeks, including the following: (1) Individualized preoperative training programme, focused on improving back and abdomen muscle strength and cardiovascular conditioning; (2) Education on operation and postoperative mobilization and rehabilitation; (3) A 200 mL protein-rich drink the evening before surgery; (4) A rehabilitation programme that aimed for discharge on day 5, intensive mobilization, and additional protein drinks post-surgery.	Usual care regarding preoperative education. Postoperatively, the patient was mobilized on the day of surgery (if possible) and trained for 30 min daily.	(1) Cost and health-related quality of life; (2) Pain (BPI) function (Roland Morris questionnaire, sit to stand, TUG, milestone achieved under hospitalization, quality of life (HRQOL: 15-D).	No difference in health-related quality of life scores were observed. The IG obtained their postoperative milestones sooner, returned to work and utilized less primary care following discharge. The intervention programme was €1625 (direct costs €494 and indirect costs €1131) less per patient than usual care costs. At operation, the IG had improved function and postoperatively reached recovery milestones faster than the CG (1–6 days versus 3–13 days *p* = 0.001). Length of stay was shorter for the IG at 5 days (range: 3–9) versus 7 days (range: 5–15) and no differences were recorded in postoperative complications, or adverse events.
Kesänen et al., 2016, 2017 [27,28] (n = 100)	Surgery for spinal stenosis. At baseline: IG: n = 50 CG: n = 50	Patients received an empowering telephone discourse based on scores from the KNOWBACK test. Items in the test included bio-physiological, functional, social, ethical and financial.	Usual care regarding preoperative education.	(1) Patient knowledge; (2) Anxiety (STAI-S), HRQoL (RAND-36), Disability (ODI), Pain (VAS).	At baseline, there was no difference in the knowledge level of the study groups. At admission, the knowledge level was significantly higher in five or six dimensions of empowering knowledge in the IG compared group to the CG. During the 3 and 6-month follow up, the knowledge level within the study groups remained stable. In the IG, a significant reduction in anxiety was noted after the intervention, whereas in the CG, anxiety reduced only after surgery. In both groups, a significant improvement in HRQoL, disability, and pain was noticed at the 6-month follow up, but there were no between-group differences.
Lee et al., 2018 [29] (n = 86)	Lumbar spinal surgery (fusion and decompression). IG: n = 43 CG: n = 43	A patient booklet covering the following topics: (1) Introduction to diseases (spinal structure, common types of spinal disease, two types of lumbar surgery and anaesthesia); (2) Introduction to the operative environment (examinations before surgery, location of the operative environment, major areas and the usual setting); (3) Surgical procedures (when and what will be arranged before entering the operative room as well as in the operative room, how the surgeons and nurses maintain cleanliness, what the surgeons and nurses will do after surgery, notes for family members and notes in recovery room); (4) Postoperative care (how to care for wounds, drainage tube, and catheter, how to correctly logroll a patient; how to correctly use collars and back pads; and precautions).	Usual care regarding preoperative education.	Anxiety (STAI), pain (VAS), patient monitors for physical indicators (cortisol levels through saliva, blood pressure, heart rate and respiration rate).	The adjusted anxiety and pain levels were significantly lower for the IG: mean STAI scores were 52.67 at baseline and 47.54 at 30 min before surgery (*p* < 0.001); mean pain scores were 6.07 at baseline and 5.28 on day after surgery (*p* < 0.001).
Lindback et al., 2017 [26] (n = 197)	Surgery for disc herniation, spinal stenosis, spondylolisthesis, or degenerative disc disease. At randomization: IG: n = 99 CG: n = 91	Pre-surgery physiotherapy twice a week for 9 weeks, including the following: (1) Physiotherapy according to a treatment-based classification; specific exercises and mobilization, motor control or traction; (2) Individualized supervised exercise programme; (3) Behavioral approach to reduce fear avoidance and increase activity level.	Usual care regarding preoperative education.	Function and activity limitation (ODI), health related quality of life (SF-36 and EQ-5D) pain intensity (VAS), anxiety, depression (HADS), self-efficacy (SES), fear avoidance (FABQ-PA), physical activity and treatment effect (PGIC).	The IG demonstrated small improvements in disability, back pain, EQ-5D, EQ-VAS, dear avoidance belief questionnaire—physical activity, SES, and HADS scores and activity level after the pre-surgery intervention. However, post-surgery, the only differences between groups was a higher activity level in the IG compared to the CG.
Chuang et al., 2016 [30] (n = 64)	Anterior cervical discectomy and fusion surgery. IG: n = 32 CG: n = 32	Twenty minutes of one-to-one education on the day of surgery, provided on an iPad and a booklet. Topics included the following: (1) A thorough explanation of cervical herniation of the intervertebral disc; (2) Surgery details; (3) Key points of postoperative care.	Usual care regarding preoperative education.	Anxiety (STAI), uncertainty (MUIS) and satisfaction with pre-surgery education (5-point Likert scale).	The educational intervention was found to be more effective than conventional care in reducing patient’s anxiety and uncertainty (*p* < 0.005). Patients were more satisfied in the IG due to a more holistic approach to individual health.

IG = intervention group; CG = control group; NPRS = numeric pain rating scale; ODI = Oswestry disability index; QALY = quality-adjusted life years; EQ-5D = EuroQol 5D; TUG = timed up and go; BPI = brief pain inventory; HRQOL = health related quality of life; STAI = state-trait anxiety inventory; HADS = hospital anxiety and depression scale; SES = self-efficacy scale; FABQ-PA = fear avoidance belief questionnaire—physical activity; PGIC = patient global impression of change; MUIS = Mishel uncertainty in illness scale.

**Table 4:** Cochrane risk-of-bias scores.

Study	Bias						
	Random Sequence Generation	Allocation Concealment	Blinding of Participants and Personnel	Blinding of Outcome Assessment	Incomplete Outcome Data	Selective Reporting	Other Bias
Rolving et al. [20]	+	−	−	−	+	+	−
Louw et al. [23]	+	+	−	−	+	+	−
Nielsen et al. [25]	+	+	−	−	−	+	+
Lindbäck et al. [26]	+	+	−	+	−	+	−
Kesänen et al. [28]	+	+	+	+	+	+	−
Lee et al. [29]	+	+	−	−	+	+	−
Chuang et al. [30]	+	−	−	−	+	+	+

+ low risk of bias; − high risk of bias.
